# Functional *in silico* analysis of human tyrosinase and OCA1 associated mutations

**Published:** 2020-08-24

**Authors:** Milan Patel, Yuri Sergeev

**Affiliations:** National Eye Institute, National Institutes of Health, Bethesda, MD 20892, USA

**Keywords:** human tyrosinase, genetic mutations, oculocutaneous albinism 1, molecular modeling, classification of disease-causing mutations

## Abstract

Oculocutaneous albinism type 1 (OCA1) is an autosomal recessive disorder caused by mutations in the tyrosinase gene. OCA1 exists in two forms: OCA1A and OCA1B. OCA1A is caused by a full loss of the human tyrosinase protein (Tyr), leading to an absence of pigment in skin, hair, and eyes, while OCA1B has reduced Tyr catalytic activity and pigment. The current understanding of the disease is hampered by the absence of information regarding the alterations of protein structure and the effects leading to either form of OCA1. Here, we used computational methods to find a general mechanism for establishing this link. Tyr and mutant variants were built through homology modeling, glycosylated *in silico*, minimized, and simulated using 100 ns molecular dynamics in water. For OCA1B mutants, cavity size is linked to ΔΔG values for mutants, suggesting that partial loss of Tyr is associated with the destabilizing effect of the EGF-like domain movement. In OCA1A, active site mutation simulations indicate that the absence of O_2_ leads to protein instability. OCA1B mutants are described in severity by the size of the cavity within the EGF–Tyr interface, while active site OCA1A mutants are unable to fully coordinate copper, leading to an absence of O_2_ and Tyr instability. In patients with known genotypes, free energy changes may help identify the severity of the disease by assessing either the allosteric effect of the EGF-Tyr cavity in OCA1B or the active site instability in OCA1A.

## Introduction

Human tyrosinase is a type 1 trans-membrane and copper-containing glycoenzyme that catalyzes the rate-limiting step of melanin pigment production in melanosomes.^1,2^ Mutations in the tyrosinase gene (*TYR*) can lead to oculocutaneous albinism Type 1 (OCA1), an autosomal recessive disease. Phenotypic signs of OCA1 include the absence (Type A) or reduction (Type B) of pigment in skin, hair, and eyes. OCA1A is associated with a complete loss of tyrosinase function, while OCA1B exhibits reduced tyrosinase enzymatic activity.^3^ Nearly 350 *TYR* mutations have been identified as causes of OCA1 in patients with established diagnosis, 77% of which are missense mutations (OCA1A – 67% and OCA1B – 33%).^4^

While recent attempts at crystallography of human tyrosinase have been unsuccessful,^5^ the crystal structure of human tyrosinase-related protein 1 domain (residues 1–537) was determined at 2.3 Å resolution, and can be used as a reference for modeling intra-melanosomal domains of mammalian tyrosinases due to their 40–50% sequence identity.^6,7^ The active site contains four α-helices containing six His residues that coordinate two zinc ions involved in the catalytic mechanism of Tyrp1. These zinc ions are partially stabilized by one water molecule between them. Crystal structures of bacterial and fungal tyrosinase species are readily available; however, in crystallographic studies, the majority of protein structures did not demonstrate protein enzymatic activities, indicating possible differences from a protein native state. Also, a human tyrosinase structure is needed to answer OCA1 mutation-related questions.

Tyr is an N-linked glycoprotein that relies on glycosylation for its proper folding.^8,9^ Glycosylation stabilizes human Tyr during the process of translocation from the endoplasmic reticulum (ER) to the cytoplasm of the melanocyte.^4^ OCA1A mutants have been shown to destabilize the Tyr structure, resulting in a loss of enzymatic activity and proteolytic degradation. OCA1B also results in structural destabilization, but to a lesser degree, meaning the mutant appears to produce lower levels of melanin in melanocytes compared to the wild type.^4,10^ The unfolding mutation screen (UMS) was developed in order to understand and evaluate the effect of missense mutations on protein folding and thermodynamic stability.^11^ This program uses protein unfolding curves and thermodynamic changes in Gibbs free energy (ΔΔG) to calculate propensities of mutations in global mutagenesis. The results are then projected onto a protein model to highlight the critical residues and regions of structural importance to the protein.

While the difference in phenotype between OCA1A and OCA1B have been identified, the difference in the molecular mechanism leading to these results have yet to be discovered. Here, we used computational methods to find a general mechanism for establishing a link between mutation changes at the Tyr protein level and OCA1 disease phenotypes. Tyr and mutant variants were built through homology modeling, glycosylated *in silico*, minimized, and simulated using 100 ns molecular dynamics in water. For OCA1B mutants, cavity size is linked to free energy changes for mutants, suggesting that partial loss of Tyr in OCA1B is associated with the destabilizing effect of the EGF-like domain movement. In OCA1A, active site mutation simulations demonstrated that the absence of O_2_ in an active site causes the inability to fully coordinate copper atoms and leads to Tyr protein instability. In patients with known phenotypes, ΔΔG values produced in our work may help identify the severity of OCA1 by assessing either the allosteric effect of the EGF-Tyr cavity in OCA1B or the active site instability in OCA1A.

## Material and methods

### Molecular modeling

A human homology model of the intra-melanosomal domain of human tyrosinase (TyrD, residues 19–469) was built, and refined using 2 ns molecular dynamics (MD). Ion concentration was added as a mass fraction with 0.9% NaCl used. Simulation temperature was set to 298 K with a water density of 0.997 at pH 7.4. The cell size extended to 10 Å beyond each side of the protein in the shape of a cube with dimensions 92.9 Å x 92.9 Å x 92.9 Å. Each mutation was run through molecular dynamics in YASARA using an AMBER03 forcefield, with a timestep of 5 fs. Tyr was run through the Unfolding Mutation Screen, and the atomic model was uploaded to the ocular proteome website (https://neicommons.nei.nih.gov/#/proteome).^11,12^ Later, the structure of tyrosinase was glycosylated *in-silico* as follows. The OCA1B PDB files were glycosylated at five biologically likely Asn sites (N111, N161, N230, N337, and N371) using the online web tool Glycam-Web (http://glycam.org). The exact sugar composition in each N-glycosylation site is not known; the most biologically common eukaryotic core glycan was chosen and attached to the site: (N-acetylglucosamine, β-D-mannose, α-D-mannose).^13,14^ Using the Build Structure function in UCSF Chimera,^15^ bonds were added between N’s Nδ2 atom and N-acetylglucosamine’s C1 atom. The O1 and HO1 atoms on N-acetylglucosamine and HD21 atom on N were deleted.

### Mutant modeling

Eleven OCA1A (H180N, H180R, H202R, H202Q, H211R, H363T, H363R, H363Y, H367R, H367Y, H390T) and seven OCA1B (W39R, R77G, K142M, S323R, T325A, M370V, V393D) mutations were generated using the Edit > Swap > Residue function on the Tyr PDB file in YASARA (http://www.yasara.org/). Tyrosinases are synthesized in the endoplasmic reticulum (ER) and function in melanosomes at two different pH conditions, pH 7.4 and pH 5.0, respectively.^16^ Therefore, the mutations were simulated in a water box using the YASARA runfast.mcr macro, with OCA1A at pH 7.4 (to replicate the ER environment) and OCA1B at pH 5.0 (to replicate the melanosome environment).^17,18^ The simulation was run as described in the molecular modeling methods section. PDB files were created at 0 ns after minimization and at 100 ns.

### Active site alterations

Tyrosinase and tyrosinase-related proteins each have coordinating molecules within the active site.^13^ In order to define their role in tyrosinase function, the Tyr and OCA1A mutant structures were simulated without the dioxygen molecule. The dioxygen that binds copper within the active site were deleted from the PDB file. After deletion, each structure was energy-minimized in a water box using the AMBER14 force field, and subsequently run through molecular dynamics. The same parameters as the original mutants were used.

### Structure comparison

Tyr and OCA1 mutant structures were compared in Chimera.^15^ Structures were overlaid onto one another and the RMSD was calculated using the MatchMaker function. The best-aligning pair of chains between reference and match structure was chosen for chain pairing. The Needleman-Wunsch algorithm using BLOSUM-62 was used for calculation. Matching was iterated by pruning long atom pairs until no pair exceeded 2.0 angstroms in order to remove far apart residues on the “match list” used to superimpose the structures.

### Accessible area and cavity analysis

Tyr and all OCA1B mutant structure simulations were analyzed at 0 ns and 100 ns using MOLE 2.5 for cavities within the protein. Loading a protein structure leads to automatic detection of cavities based on an algorithm.^19^ The default parameters for this algorithm are 3.00 Å for the probe radius, 1.25 Å for the interior threshold, and 5.00 Å for the minimum depth. These parameters were used in the calculation of cavities for the Tyr and OCA1B mutants. The three largest cavities of each structure were summed and used for analysis, assumed to be a reasonable approximation for the total cavity volume in each protein structure. For mutant variants, the effect of MD simulations was estimated as a difference between parameter changes in protein structure (mut) at 100 ns and 0 ns structure (wt). The change in solvent-accessible surface area (SA) over 100 ns between structures was described as ΔΔSA = ΔSA_mut_ - ΔSA_wt_. The change in solvent-accessible volume (V) over 100 ns between structures was described as ΔΔV = ΔV_mut_- ΔV_wt_. The change in cavity volume (V_cav_) over 100 ns between structures was described as ΔΔV_cav_ = ΔV_cav_mut_ - ΔV_cav_wt_.

### Unfolding mutation screen

Global mutagenesis was conducted on Tyr, and each mutant was characterized by a thermodynamic change in Gibbs free energy (ΔΔG). These values were calculated using the semi-empirical method (FoldX) and standardized on a 0–1 scale, named the unfolding parameter, known as the fraction of protein in the unfolded state.^11,20^ This parameter described the predicted effect of each mutation as weak (0–0.19), moderate (0.2–0.8), or severe (0.81–1). The Unfolding Mutation Screen (UMS) also outputted a foldability parameter to show critical residues in protein folding. This parameter is a sum of severity-weighted unfolding propensities for the 20 mutations generated at a specific residue. Residues with the highest foldability were considered critical for protein folding.^11,14^

### Statistical analysis

Within the UMS pipeline, a procedure called “internal control” verifies the quality of the protein structure by mutating each residue from the protein sequence to itself.^11^ The ΔΔG values of each identity mutation are calculated and then converted to unfolding propensities. The quality of the protein model is then determined by calculating the mean, standard deviation, p-value, and 95% confidence interval for those propensities. Each unfolding propensity should have a value of 0.5. Statistical significance of OCA1 structural parameters were assessed using Pearson’s correlation coefficient and an adjusted R-squared value. The Pearson’s correlation coefficient formula is:

$r=\frac{n\left(\sum xy\right)-\left(\sum x\right)\left(\sum y\right)}{\sqrt{\left(n\sum x^{2}-{\left(\sum x\right)}^{2}\right)\left(n\sum y^{2}+{\left(\sum y\right)}^{2}\right)}}$, where n is the number of points. The adjusted R-squared formula is:

$R_{adj}^{2}=1-\left[\frac{\left(1-R^{2}\right)(n-1)}{n-k-1}\right]$, where n is the number of points and k is the number of independent repressors.

## Results

### Homology model of Tyr

**Figure 1:**
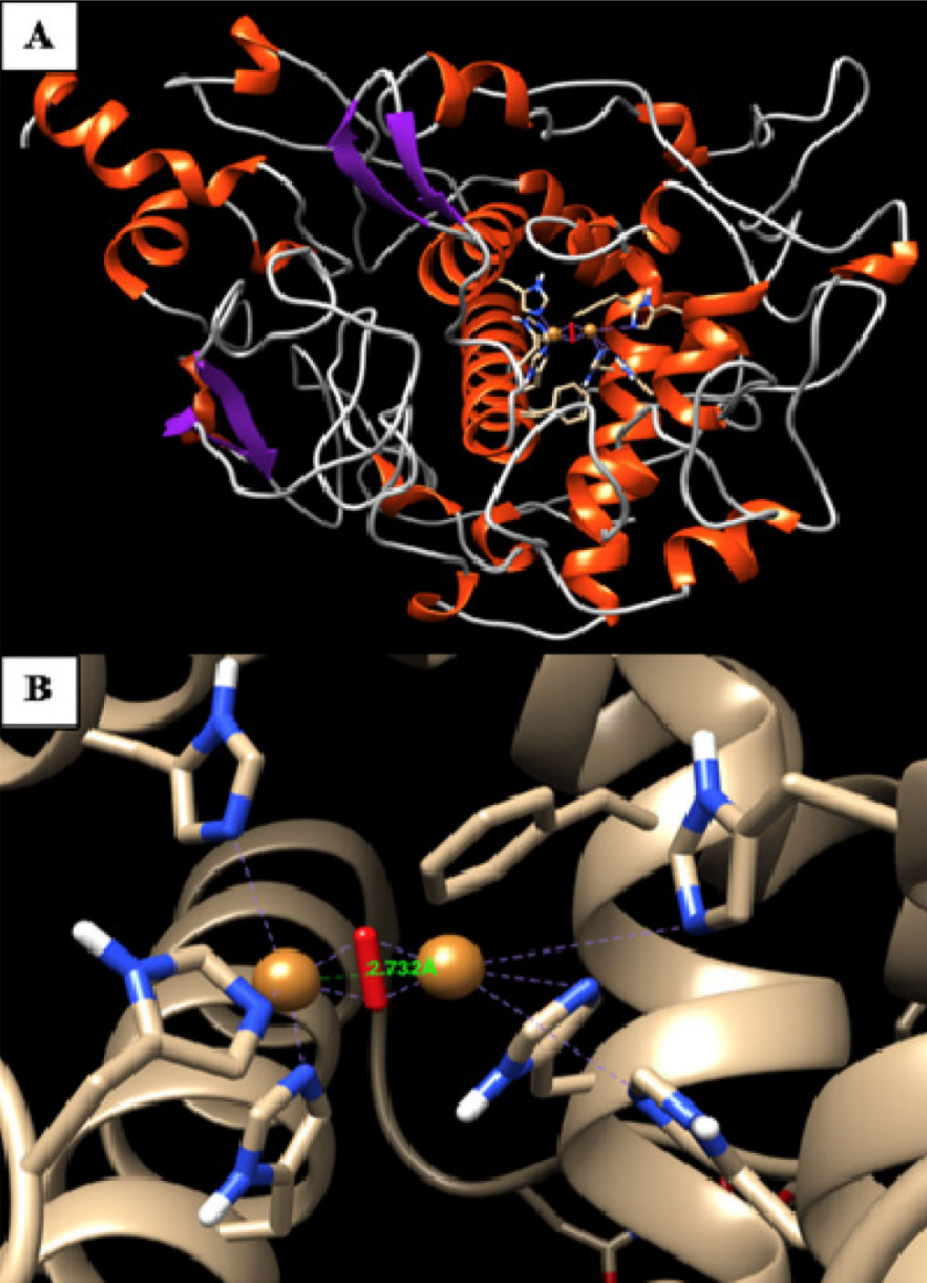
(A) Tyr homology model colored by secondary structure. α-Helices (and copper atoms) are highlighted in orange, beta sheets are highlighted in purple, coils are highlighted in gray. (B) The ribbon structure of the Tyr active site with coordinating copper His residues displayed. The distance between the copper atoms is 2.7–2.8 Å, and the O_2_ molecule is located between them, stabilizing the two atoms.

**Figure 2:**
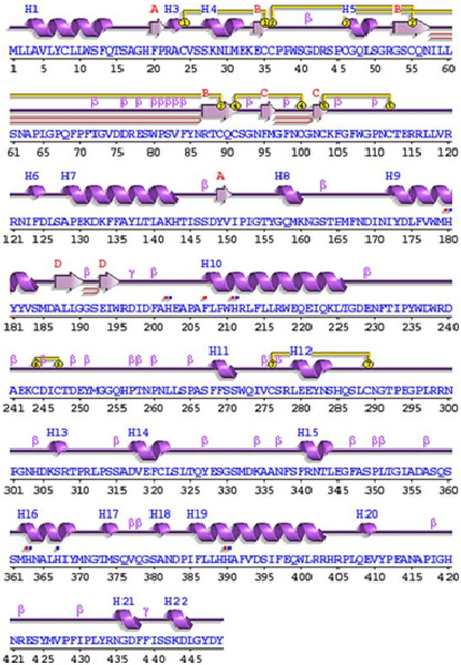
Sequence of the intra-melanosomal domain of human Tyr with respective secondary structure elements labeled. Obtained using the pdbsum generate server (https://www.ebi.ac.uk/thornton-srv/databases/pdbsum/Generate.html).

Tyr homology model contains 12 alpha helices of at least one turn, four beta sheets, and seven disulfide bonds. The first five disulfide bonds are situated in the EGF-like domain, a repeat found in many extracellular and cell surface proteins (Figure 1A), (Figure 2). This domain is also found in homologous tyrosinases, Tyrp1 and Tyrp2. While the typical EGF-like repeat contains three disulfide bridges, the Tyr structure contains two more within the same region, providing more stability.

**Figure 3:**
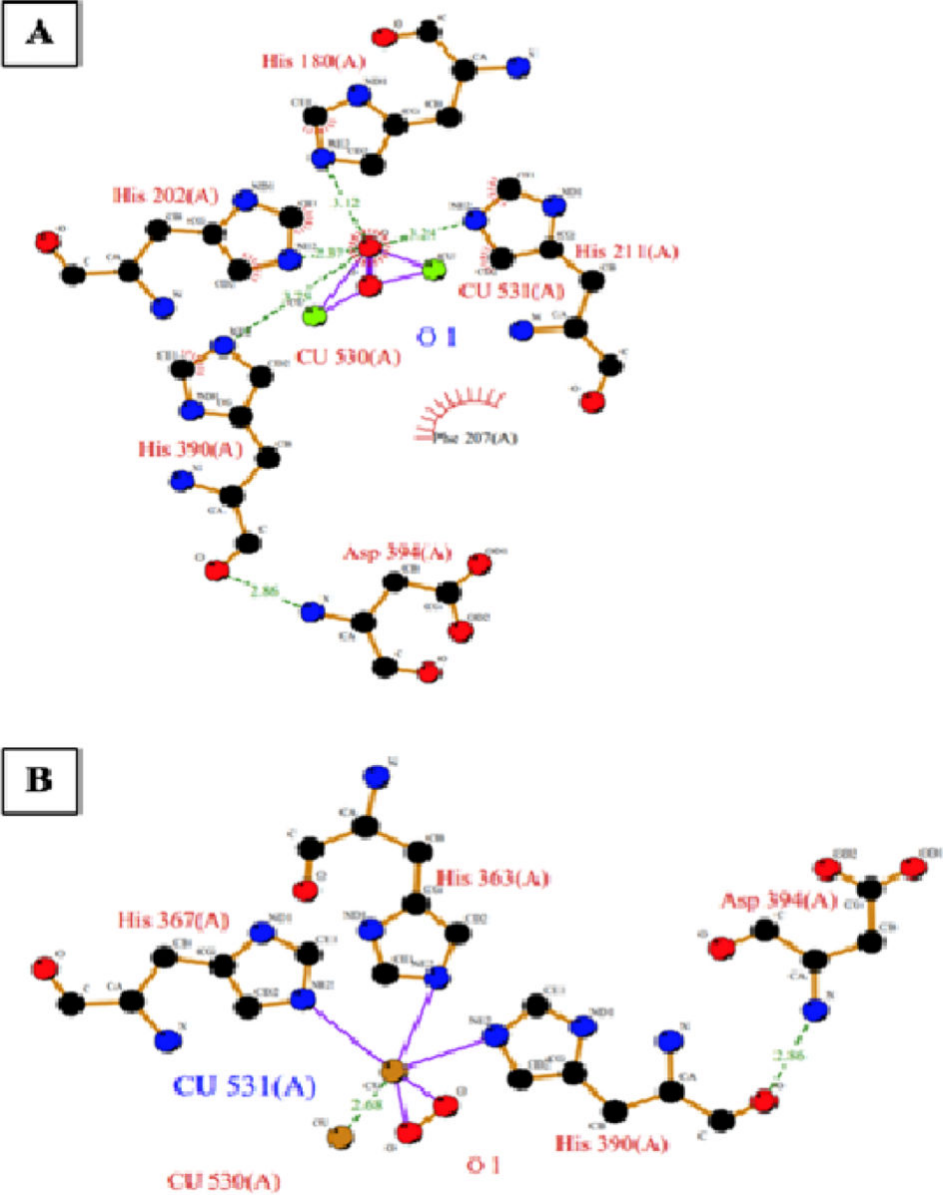
Interactions within the active site. A. Histidine interactions with CuA. B. Histidine interactions with CuB. Obtained using the pdbsum generate server (https://www.ebi.ac.uk/thornton-srv/databases/pdbsum/Generate.html).

The active site of Tyr contains a bundle motif containing four α-helices (H9, H10, H16, H19) surrounding a copper-dioxygen-copper complex involved in the catalytic mechanism (Figure 1B), (Figure 3).^1^ Within the active site are six His residues that coordinate the two coppers, which are held together 2.7–2.8 Å apart. The first copper, CuA, is coordinated by H180, H202, and H211 (Figure 3A). H180 is in α-helix H9, H211 is located within α-helix H10, and H202 is located in between α-helix H9 and α-helix H10. The second copper, CuB, is coordinated by H363, H367, and H390 (Figure 3B). H363 and H367 are situated within α-helix H16, and H390 is situated within α-helix H19.

### Structural changes caused by OCA1B mutations

In order to define key characteristics of OCA1 and molecular differences between OCA1A and OCA1B, eight OCA1B mutants and eleven active site His OCA1A mutants were analyzed. The ΔΔG value and unfolding parameter were calculated for each OCA1B mutant using UMS (Table 1).

Foldability parameters were listed for each residue of interest, highlighting each residue’s importance to structural and thermodynamic stability (Table 2).

For these residues, R77, M370, V393, and R402 were predicted to have the greatest destabilizing effect with ΔΔG = 2.1–5.7 kcal/mol. K142 is in α-helix H7, located away from the active site. S323 and T325 are residues in the coil between α-helix H14 and α-helix H15, located on the periphery of the protein, and do not contribute to the secondary/tertiary structure of Tyr. R77 is located within the beta hairpin of beta sheet B. M370 is located one residue after trans-membrane α-helix H16. V393 and R402 are both located within trans-membrane α-helix H19. W39 is located within the EGF-like motif (Figure 2).

**Figure 4:**
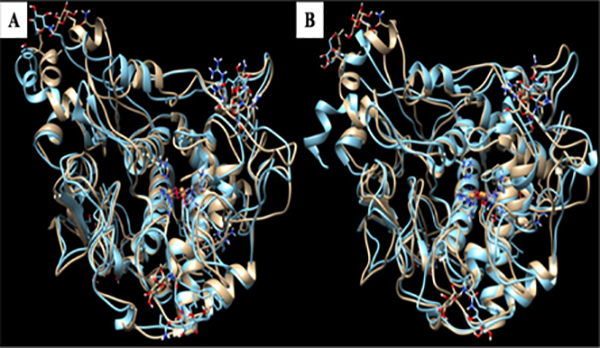
The superposition of Tyr native structure and K142M and R77G mutant variants obtained after 100 ns MD run. (A) The superposition of the protein atomic structures of native Tyr (bronze) and the K142M mutant variant are overlaid onto one another using MD at 100 ns. Both proteins are represented by ribbon structures. There were no visible differences found in regions of importance. The RMSD across pruned atom pairs was 1.2 Å. (B) The ribbon structures of Tyr (bronze) and R77G (cyan) are overlaid onto one another at 100 ns. There were no visible differences found in regions of importance. The RMSD across pruned atom pairs was 1.3 Å.

Mutant structures were overlaid onto the Tyr structure at 100 ns to identify visible structural changes (Figure 4). No visible differences were found within the transmembrane α-helices, active site, EGF-like domain, or glycan-rich regions. The ΔΔG value of unfolding did not correlate with differences in secondary or tertiary structure in OCA1B mutants. K142M, a stable yet non-native mutant, and R77G, the most unstable mutant, exhibited similar minor differences in random loop fluctuations, but no difference in Tyr regions of importance (EGF-like domain, active site, and secondary structure). The structure comparison of Tyr and K142M yielded an RMSD across pruned atom pairs of 1.2 Å and an RMSD across all atom pairs of 2.4 Å. The structure comparison of Tyr and R77G yielded an RMSD across pruned atom pairs of 1.3 Å and an RMSD across all atom pairs of 3.3 Å.

**Figure 5:**
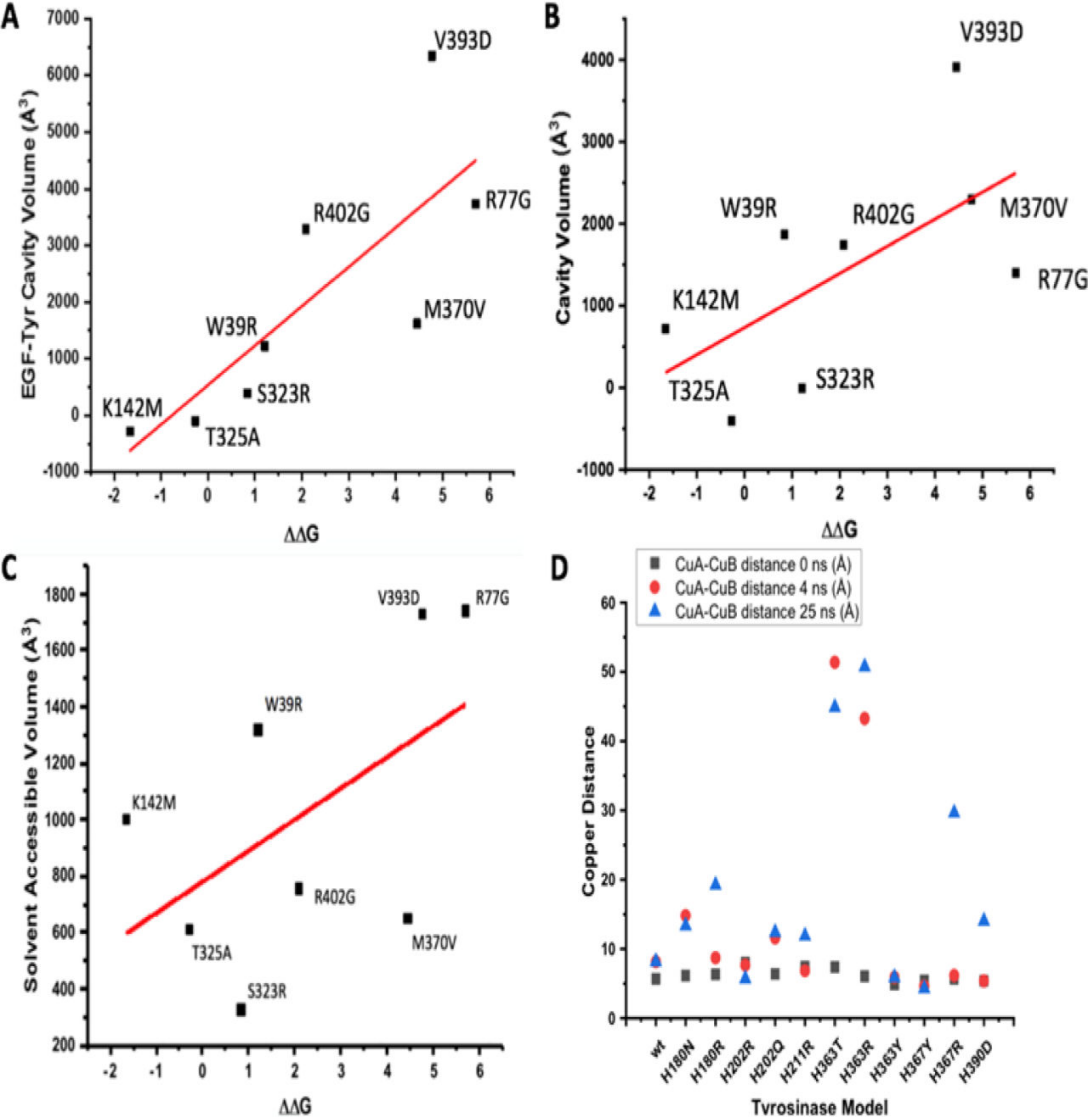
Changes in cavity volume, solvent-accessible area and volume, and inter-atomic copper distances for OCA1 mutations. (A) Graph of ΔΔG vs change in EGF–Tyr interface cavity volume from difference at 0 ns and 100 ns (ΔΔV_cav_) for OCA1B mutations. Pearson’s coefficient of 0.80. Adjusted R-square value of 0.57. Regression equation: ΔΔ*V*_*cav*_ = 749.1 x ΔΔ*G* + 423.0. (B) Graph of ΔΔG vs change in solvent-accessible volume from difference at 0 ns and 100 ns for OCA1B mutations. Pearson’s coefficient of 0.55. Adjusted R-square value of 0.18. (C) Graph of ΔΔG vs change in cavity volume from difference at 0 ns and 100 ns for OCA1B mutations. Pearson’s coefficient of 0.63. Adjusted R-square value of 0.30. (D) Copper distances measured at 0 ns, 4 ns, and 25 ns for Tyr and each His active site mutation. For mutants with copper distances <10 Å at 25 ns, it is important to note that the copper initially coordinated by two His residues had lost all coordination at 25 ns.

Solvent-accessible volume and cavity volume within the protein were collected for each OCA1B mutant variant in MD simulations in order to identify a link between these parameters and Gibbs free energy changes. Solvent-accessible volume was calculated by YASARA. Calculated by Mole 2.5, total cavity volume was determined as the sum of the three largest cavities within the protein. Over 100 ns, the difference in solvent-accessible volume increased directly for each mutant with the ΔΔG value (Table 1). The ΔΔSA value compared to ΔΔG had a Pearson’s Coefficient of 0.55, indicating a low association between the severity of the mutation as described by ΔΔG and the total surface of the protein (Figure 3B). The ΔΔV value compared to ΔΔG had a Pearson’s Coefficient of 0.63, indicating that the total volume of the protein increases with the severity of the mutation (Figure 5C).

While OCA1A is characterized as a complete loss of Tyr function, OCA1B exhibits reduced enzymatic activity. Tyr seems to complete the folding process; however, mutations within the protein result in a partial loss of activity. According to our data, once OCA1B mutants had completed the folding process, the protein structure began to slowly expand, creating cavities that channeled water molecules to the interior of the protein. The most severe mutations of Tyr resulted in large cavities of up to 6500 Å^3^ of a difference compared to the native Tyr (Figure 5C). Cavities were then analyzed for location commonality in regard to secondary structures, the EGF-like domain, and the active site.

**Figure 6:**
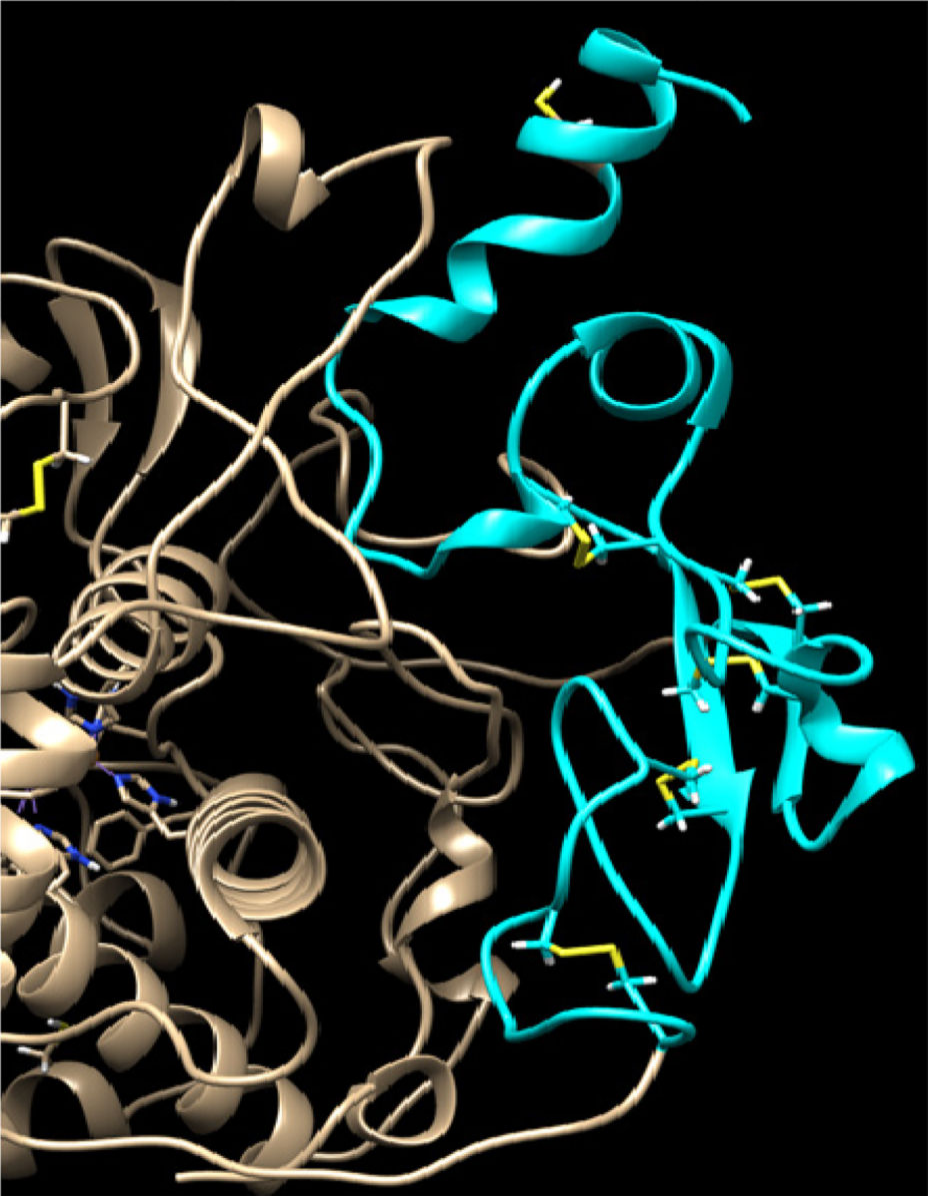
The cavity localized between tyrosinase and EGF-like domains. The EGF-like domain is highlighted in blue and the rest of Tyr is colored bronze. The disulfide bridges are displayed.

Large Tyr cavities were found near the EGF-like domain, which is located within the first 100 residues of the protein. The EGF-like domain is a cysteine-rich region that contains five disulfide bridges (Figure 6).^21^ The hydrophobic cavity between the EGF-like domain and the rest of the Tyr domain (EGF–Tyr interface) contains many large aromatic residues (F98, F105, F176, W210, F429, Y433, and F438). This interface was monitored to assess destabilization in Tyr mutants in OCA1B (Table 3). The EGF-Tyr interface volume was 422 Å^3^ at 100 ns for native Tyr. Mutants with ΔΔG<0 contained cavities with decreased size compared to Tyr within the cysteine-rich domain. Mutants with ΔΔG>0 contained significantly larger cavities within the EGF-Tyr interface, leading to destabilization of the protein (Figure 5A). A strong correlation was found between the two parameters. Cavities within or near the active site show no correlation with ΔΔG values and, therefore, are not listed.

Large cavities within the EGF-Tyr interface suggest it to be a source for destabilization in OCA1B mutants. At 100 ns, the cavity encompassing the EGF-Tyr interface had perforated throughout the protein, ultimately affecting the active site in severe mutations. The native Tyr and weak mutants did not display these large cavities.

### Human tyrosinase active site

Within the active site of tyrosinase there is a bundle of four alpha helices that contain six copper coordinating histidine residues. The two copper atoms are stabilized and tightly held together (2.7–2.9 Å) by two oxygen atoms (Figure 1B). Mutations in copper coordinating histidine residues could cause a partial or complete loss of protein activity.^4^ In order to find the mechanism leading to protein instability, we analyzed albinism causing genetic mutations localized in histidines coordinating copper within the active site of Tyr. The foldability parameter for each active site histidine residues is presented in Table 4. For these residues, mutations to H202 and H211 were predicted to have the greatest destabilizing effect. UMS calculations for individual mutations predicted significant changes causing a loss of protein stability or a stabilizing effect leading to a non-native stable structure (Table 5).

**Figure 7:**
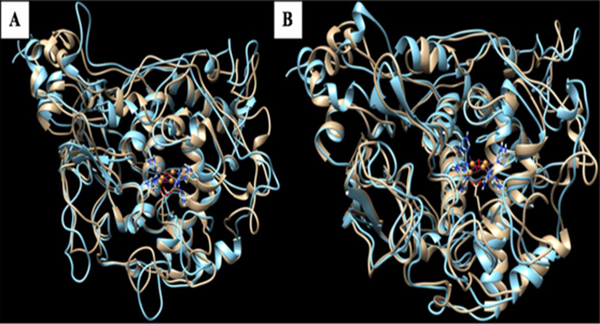
The superposition of Tyr native structure and H363T and H211R mutant variants were obtained after 100 ns MD run. (A) The ribbon structures of Tyr (bronze) and H363T (cyan) are overlaid onto one another at 100 ns. The RMSD across pruned atom pairs is 1.1 Å. (B) The ribbon structures of Tyr (bronze) and H211R (cyan) are overlaid onto one another at 100 ns. The RMSD across pruned atom pairs is 1.2 Å.

The effect of mutations was simulated using MD. Each mutant variant was generated as described in the Methods section. After simulation of each OCA1A mutant was completed, the differences of the molecular-accessible volume and surface area between 0 ns and 100 ns were recorded in order to assess structural changes (Table 5). No correlation was found between mutation severity and ΔΔV (Pearson’s Coefficient = −0.51) or ΔΔSA (Pearson’s Coefficient = −0.62), suggesting the correlation between ΔΔG and OCA1B mutant expansion might not apply to active site His mutants. No significant visual differences were found between Tyr and the OCA1A mutants, and there was no correlation with the ΔΔG values (Figure 7). The structure comparison of Tyr and H363T, the most stable OCA1A mutant, produced an RMSD across pruned atom pairs of 1.1 Å and an RMSD across all atom pairs of 3.1 Å at 100 ns. The structure comparison of Tyr and H211T, the most severe OCA1A mutant, produced an RMSD across pruned atom pairs of 1.2 Å and an RMSD across all atom pairs of 3.1 Å at 100 ns.

### Effect of O_2_ in histidine active site mutant function in OCA1A

**Figure 8:**
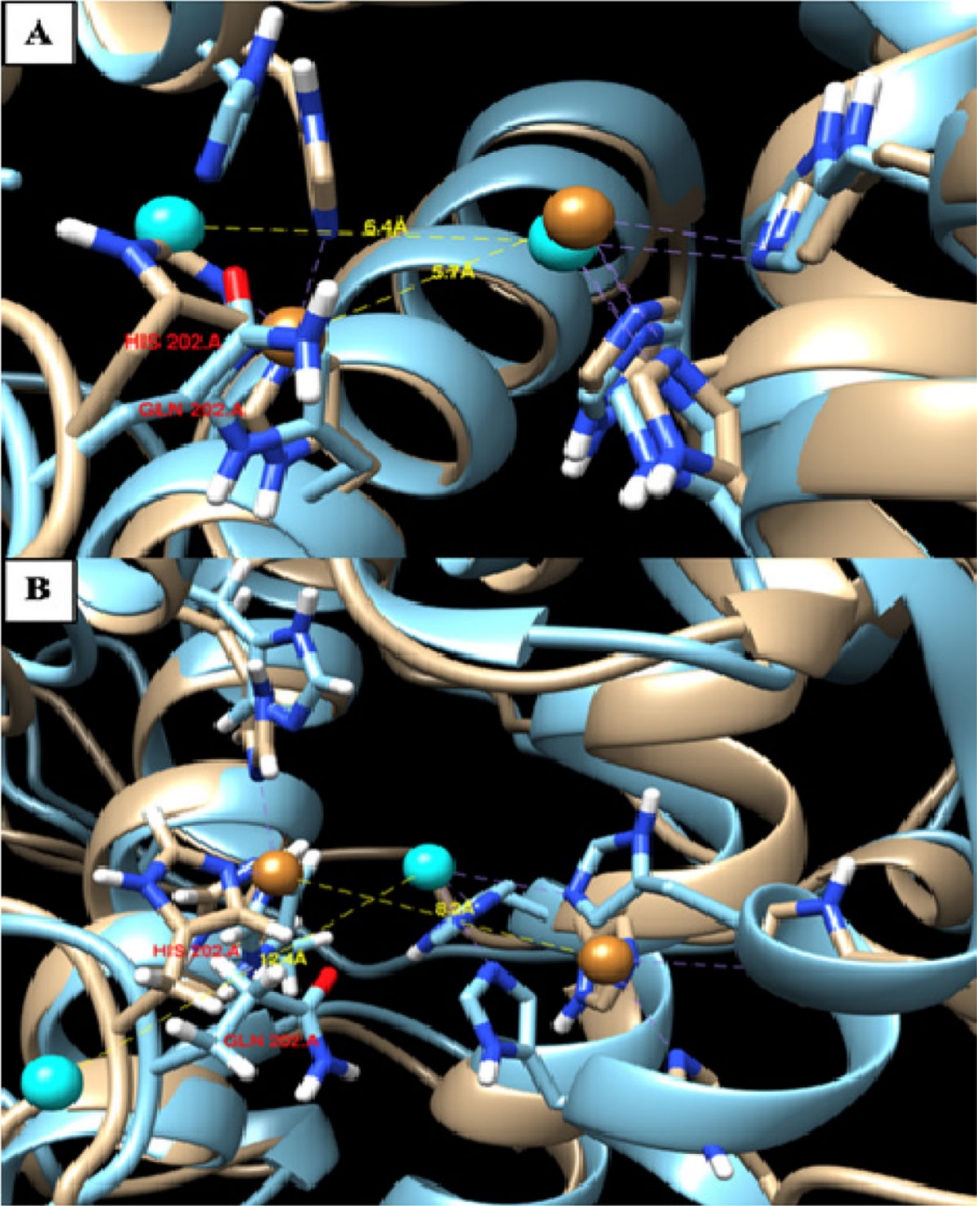
The superposition of Tyr without O_2_ and H202Q without O_2_. (A) The ribbon structures of the Tyr (bronze) and the H202Q mutant (cyan) are overlaid onto one another at 0 ns. Histidine residues required for copper coordination are shown in stick form with copper atoms in bronze for the Tyr and cyan for the H202Q mutant. The distance between the two copper atoms is 5.7 Å for the WT and 6.4 Å for the mutant. (B) The ribbon structures of the Tyr (bronze) and the H202Q mutant (cyan) are overlaid onto one another at 25 ns. Histidine residues required for copper coordination are shown in stick form with copper atoms in bronze for Tyr and cyan for the H202Q mutant. The distance between the two coppers is 8.2 Å for Tyr and 12.4 Å for the mutant. Q202 is not able to coordinate the copper ion, increasing the separation between the atoms. The active site of H202Q is larger than the WT, due to a slight unfolding of the protein.

The glycosylated Tyr and all eight OCA1B mutant simulations were run with and without O_2_-binding copper within the active site to define its role in Tyr function. After 25 ns, the copper distance for Tyr expanded to 8.2 Å. None of the mutants were located within the active site or were able to affect copper stability, so there was no major difference with the OCA1B mutants. Although the stabilizing effect of O_2_ was removed, three His residues still coordinated each copper and held them within the active site. The dioxygen molecule bound to both coppers, thought to be integral to protein stability, was removed from Tyr and the active site mutations. Simulations for all models without dioxygen were run for 25 ns, and the copper distance was measured at 0 ns, 4 ns, and 25 ns (Table 6). For Tyr without O_2_, the copper atoms separated to 8.2 Å at 4 ns and were held stable for the rest of the simulation. For each active site His mutant, one of the two coppers lost coordination. The copper coordinated by two His residues was no longer held in place in the active site, and it either moved out and away from the two His residues or out of the active site entirely, indicating that the absence of O_2_ leads to protein instability in active site His mutations (Figures 5D & 8). In those mutants, the other copper was coordinated by three His residues and did not exhibit any signs of loss of coordination, keeping its relative position throughout the simulation. These results suggest that OCA1A mutant misfolding could be associated with protein instability within the active site.

## Discussion

Oculocutaneous albinism type 1 (OCA1) is an autosomal recessive disorder caused by mutations in the tyrosinase gene. OCA1 exists in two forms: OCA1A and OCA1B. OCA1A is caused by a full loss of the human tyrosinase protein (Tyr), leading to an absence of pigment in skin, hair, and eyes, while OCA1B has reduced Tyr catalytic activity and pigment. In this work, Tyr and mutant variants were built through homology modeling, glycosylated *in silico*, minimized, and simulated using 100 ns molecular dynamics in water. For OCA1B mutants, cavity size is linked to ΔΔG values for mutants, suggesting that partial loss of Tyr in OCA1B is associated with the destabilizing effect of EGF-like domain movement. In OCA1A, active site mutation simulations indicate that the absence of O_2_ leads to protein instability. OCA1B mutants are described in severity by the size of the cavity within the EGF-Tyr interface, while active site OCA1A mutants are unable to fully coordinate copper, leading to an absence of O_2_ and Tyr instability. In patients with known genotypes, ΔΔG values may help identify the severity of OCA1 by assessing either the allosteric effect of the EGF-Tyr cavity in OCA1B or the active site instability in OCA1A.

Many conserved regions exist in the tyrosinase family proteins, one of which is an EGF-like domain.^21^ This motif is comprised of a cysteine-rich region that contains five disulfide bridges, assisting with protein stability. Although the majority of OCA1B mutations are not located within the EGF-like motif, they seem to have an allosteric affect that slowly decreases Tyr function. When ΔΔG<0, the cavity between the EGF-like domain and the Tyr domain decreases compared to Tyr. The mutant cavity size increases directly with ΔΔG values, as the EGF-like domain moves away from the Tyr domain. Due to the hydrophobic character of the cavity, the continuous expansion and channeling of water is thermodynamically unfavorable and is accompanied by a negative enthalpy and entropy change. This leads to destabilization of the Tyr-EGF interface that propagates throughout the entire domain, resulting in an unstable protein. This gradual process can contribute to a decrease in Tyr activity and OCA1B. Simulation results indicate that the mutants only partially denature, meaning they still might conserve some functionality. Active site histidine mutations show no correlation with EGF-like domain movement and do not display signs of unfolding.

The effect of cavity formation due to mutation changes, in part, could be interpreted at the level of interatomic interactions. Indeed, mutation K142M results in the removal of two hydrogen bonds. The first is between hydrogen 1HZ and oxygen OD2 atoms of K142-1HZ and D174-OD2, and is 2.0 Å. The second is between K142-2HZ and D174-OD1, and is 2.7 Å. These bonds are located on the surface of the protein and, in the K142M mutation, M142 rotates towards the interior. W39 is located between R52 and R43, and the W39R mutation can result in repulsive interactions between three consecutive arginine residues. W39R is 4.1 Å away from R52 and 4.4 Å away from R43, leading to greater destabilization in the EGF-like region and a greater ΔG value. The effect of the M370Vmutation is less definitive. Arginine R77 forms two hydrogen bonds with aspartic acid D75. The first is between R77-1HH1 and D75-OD1, and its length is 1.7 Å. The second is between R77-HE and D75-OD2, and is 1.8 Å. The R77G mutation removes both hydrogen bondsand replaces Arg with a glycine residue. S323R and T325A do not experience much of a change in environment. R402 is located on the surface of the protein at the end of α-helix H19. The R402G mutation may affect the stability of the α-helix, though it is not shown in the simulation. V393D introduces a polar residue within the hydrophobic core and forms the hydrophobic bond D393:HD1-H389:OD1 of length 1.7 Å. The simulation does not show a change in the shape of α-helix H19; however, the D393 changes orientation from V393, which could destabilize an α-helix containing His-coordinating copper. While mutations might have an allosteric effect on the EGF-Tyr interface, large ΔΔG values can also be attributed to changes within those mutations’ environments for most cases.

There are six total His-Cu coordinating bonds in Tyr. When a His residue is mutated within the active site, five other His-Cu coordinating bonds are still able to keep the protein intact. After 100 ns of His active site OCA1A mutant simulations, there was no evidence of denaturation or significant structural differences between the mutants and Tyr. This suggests that His active site mutations are destabilized prior to protein folding, since OCA1A mutants are known to be denatured. Parameters derived from the UMS calculation indicate that OCA1A active site histidine residues are not necessarily critical for protein folding; however, this parameter does not account for the loss of copper coordination (Table 4). Lack of significant change in free energy suggests that active site mutations are not able to coordinate the copper before the protein folding process is completed.

The OCA1A mutant folding process may hinge on oxygen-binding copper bringing together two halves of the protein, which could build the protein catalytic site by the formation of native 4-helix bundle motif. To test protein structure stability without the oxygen, the O_2_ molecule was removed from the protein and simulated for 100 ns. Without the oxygen molecules the copper atoms are no longer held tightly together, and their separation gradually increases, thereby affecting the substrate binding site located between the α-helices and destabilizing the protein structure. For almost every mutant, the solvent-accessible surface area and solvent-accessible volume increased compared to the Tyr structure. Additionally, when the histidine residues are no longer completely able to coordinate copper with the help of dioxygen, the surface area and volume of the protein accessible to solvent begins to increase greatly in comparison to the surface area and volume of the tyrosine wild type (Tables 5, 6).

In patients with known OCA1 genotypes, computer simulations performed in our study may help identify the severity of OCA1. By assessing either the allosteric effect of the EGF-Tyr cavity in OCA1B or the active site instability in OCA1A, we can guide treatment tailored to individual patients with OCA1. In disease, this can help identify the Tyr molecular mechanism associated with partial or complete protein misfolding.

## Conclusions

From our analysis of human tyrosinase structure, we demonstrated an association between changes in mutant variants and OCA1 phenotypes. Indeed, OCA1B mutants are described in severity by the size of the cavity within the EGF–Tyr interface, while active site OCA1A mutants are unable to fully coordinate copper, leading to an absence of O_2_ and Tyr instability. In patients with known genotypes, free energy changes may help identify the severity of the disease by assessing either the allosteric effect of the EGF-Tyr cavity in OCA1B or the active site instability in OCA1A.

## Figures and Tables

**Table 1:** OCA1B mutant simulation results

Mutation	ΔΔG*	Unfolding	Predicted Effect	ΔΔV
W39R	1.2	0.88	Severe	1248
R77G	5.7	1	Severe	1670
K142M	−1.7	0.06	Severe	928
S323R	0.8	0.8	Moderate	255
T325A	−0.3	0.39	Severe	540
M370V	4.5	1	Severe	579
V393D	4.8	1	Severe	1656
R402G	2.1	0.97	Severe	685

* For mutants with ΔΔG values below 0, we assume the structures to have a more stable but still non-native structure. Therefore, they are labeled as severe

**Table 2:** Foldability parameters for OCA1B mutations

Residue	W39	R77	K142	S323	T325	M370	V393	R402
Foldability	1.83	18.95	8.79	5.75	0	15.96	14.83	12.38

**Table 3:** Size of cavity between EGF-like domain and Tyr domain for each mutant

Mutant	Tyr	K142M	T325A	S323R	W39R	R402G	M370V	V393D	R77G
Cavity (Å^3^)	422	137	320	810	1648	3709	2041	6766	4154

**Table 4:** Foldability parameters for active site mutations

Residue	H180	H202	H211	H363	H367	H390
Foldability	3.97	14.83	14.93	3.91	0.99	3.99

**Table 5:** OCA1A mutation simulation results containing oxygen binding copper

Mutation	ΔΔG*	Unfolding Parameter	ΔΔV	ΔΔSA
H180N	−0.95	0.17	563	705
H180R	2.16	0.97	1178	1020
H202R	2.81	0.99	66	49
H202Q	1.36	0.91	−294	54
H211R	9.24	1.00	−435	−376
H363T	−1.46	0.08	1255	1419
H363R	−0.80	0.21	1505	1610
H363Y	1.58	0.93	−632	−314
H367Y	0.93	0.82	−285	−36
H367R	−0.91	0.18	813	927
H390D	−0.17	0.43	−178	146

* For mutants with ΔΔG values below 0, we assume the structures to have a more stable but still non-native structure. Therefore, they are labeled as severe

**Table 6:** OCA1A mutation simulation results without oxygen binding copper

Tyrosinase Model	Copper distance 0 ns (Å)	Copper distance 4 ns (Å)	Copper distance 25 ns (Å)
Tyrosinase Wild Type	5.7	8.2	8.2
H180N	6.1	14.8	13.4
H180R	6.3	8.7	19.2
H202R	8.0	7.7	5.7
H202Q	6.4	11.6	12.4
H211R	7.4	6.8	11.9
H363T	7.4	51.4	44.9
H363R	6.1	43.3	50.8
H363Y	4.9	5.9	5.9
H367Y	5.4	4.6	4.3
H367R	5.7	6.2	29.6
H390D	5.4	5.4	14.0

## Abbreviations:

Tyr: tyrosinase
OCA1: oculocutaneous albinism 1
OCA1A: oculocutaneous albinism 1 type A
OCA1B: oculocutaneous albinism type B
RMSD: root-mean-square deviation
EGF: epidermal growth factor
EGF-Tyr cavity: a cavity in the interface between tyrosinase and EGF-like domains
MD: molecular dynamics
UMS: unfolding mutation screen
